# From exploration to co-design: Understanding and redesigning workplace mental-support services

**DOI:** 10.1371/journal.pone.0348067

**Published:** 2026-05-08

**Authors:** Hakan Kuru

**Affiliations:** 1 Faculty of Industrial Design Engineering, Human-Centered Design, Delft University of Technology, Delft, Netherlands; 2 Faculty of Sports Sciences, Department of Coaching Education, İstanbul Rumeli University, İstanbul, Türkiye; LMU München: Ludwig-Maximilians-Universitat Munchen, NEPAL

## Abstract

Workplace mental-health support services (MSS), including employee assistance programs, are widely implemented yet chronically underutilized, with uptake rates often below five percent. This persistent gap between availability and use raises critical questions about why employees do not engage with formally accessible and confidential support. Existing research has documented attitudinal, stigma-related, and organizational barriers, but offers limited insight into the behavioral mechanisms sustaining non-use in everyday work contexts. To address this, the present study explores the underutilization of within a high-tech organization in Türkiye characterized by persistently low service uptake. Guided by the Capability, Opportunity, Motivation–Behavior (COM-B) model, an exploratory qualitative study involving semi-structured interviews with 13 employees who had never accessed the available service, followed by a participatory co-design component. Reflexive thematic analysis was used to explore key barriers and facilitators across COM-B domains, while co-design activities captured employee-generated suggestions for redesigning support. The findings indicate that persistent non-use of mental-support services was not attributable to a single dominant barrier, but emerged from the combined effects of cognitive uncertainty about service relevance, structural constraints embedded in everyday work practices, and motivational tensions related to professional identity and emotional fatigue, which together reduced the likelihood that employees would initiate support-seeking. Co-design outputs translated these barriers into concrete redesign directions, including simplifying entry points, embedding support within everyday workflows, and strengthening visible organizational endorsement. By identifying how the interaction among capability, opportunity, and motivation conditions maintains non-use, this study provides a behaviorally grounded explanation for the persistent underutilization of workplace mental-support services. The findings highlight the need for organizational approaches that move beyond awareness and availability toward behaviorally aligned system design to enable meaningful employee engagement.

## 1. Introduction

Employee mental health has become a central concern for organizations seeking to sustain productivity, retention, and long-term performance. High-pressure, knowledge-intensive work environments expose employees to heavy workloads, tight deadlines, and continuous performance demands, conditions consistently associated with elevated levels of stress, burnout, and psychological strain [1,2]. In response, many organizations have invested in workplace mental support services (MSS), most commonly delivered through Employee Assistance Programs (EAPs), to provide confidential psychological support and promote employee wellbeing [3].

Despite widespread implementation, the actual use of these services remains consistently low. Across sectors and organizational contexts, annual utilization rates typically range between 3 and 8 percent of eligible employees, with some organizations reporting uptake below 1 percent [4,5]. This pattern persists even when services are free, externally provided, and formally promoted as confidential. The resulting gap between availability and use represents a well-documented paradox in workplace mental health provision.

Existing research has identified several factors associated with this paradox. At the individual level, concerns about confidentiality, anticipated stigma, and doubts about the relevance of services have been shown to discourage help-seeking in workplace settings [4,6,7]. At the organizational level, leadership signalling, cultural norms emphasizing self-reliance and uninterrupted productivity, and structural constraints such as time pressure and limited privacy shape employees willingness and ability to engage with support [8]. While these studies provide valuable insights, they have explored these factors in isolation and have focused primarily on attitudes or utilization rates rather than on the behavioral processes through which non-use is sustained in everyday work contexts.

Notably, prior research privileges the perspectives of employees who have already accessed MSS, relatively larger group of persistent non-users underexplored [4,7]. As a result, there remains a limited understanding of how everyday work practices, organizational structures, and employee decision-making processes interact to sustain non-engagement over time [1,8]. Factors such as workload rhythms, cognitive uncertainty about service relevance, personal identity, and emotional readiness are often discussed separately, rather than as interacting conditions that jointly shape help-seeking behavior [6].

Addressing these gaps requires going beyond awareness-based or attitudinal explanations toward a behavioral diagnosis of underutilization. The Capability, Opportunity, Motivation–Behavior (COM-B) model [9] conceptualizes behavior as the outcome of interacting psychological and physical capabilities, environmental and social opportunities, and reflective and automatic motivations. By emphasizing interaction rather than single-factor explanations, COM-B provides a suitable lens for examining why formally available services may remain behaviorally inaccessible in practice.

Accordingly, this study aims to identify the behavioral factors underlying the non-use of workplace mental-support (MSS) services within a high-tech organization characterized by persistently low uptake. Using an exploratory qualitative design, this study focuses explicitly on employees who had never accessed the available service. By combining semi-structured interviews with a participatory co-design component, the study explores how capability, opportunity, and motivation interact in everyday work life to maintain non-engagement, while also eliciting employee-generated ideas for redesigning support in ways that better align with organizational realities.

The remainder of the paper is structured as follows. The literature review examines workplace MSS utilization and introduces the COM-B model. The methods section describes the exploratory qualitative design. The results section presents findings organized around COM-B domains. The discussion outlines implications, limitations, and future directions. The paper concludes with key insights.

## 2. Literature review

### 2.1. Utilization of MSS in organizations

Workplace mental-support services (MSS), most commonly delivered through Employee Assistance Programs (EAPs), are widely implemented across large organizations as a strategy to support employee wellbeing and reduce productivity losses associated with stress, burnout, and mental health problems [3,10]. In many high-income countries, organizational adoption of such services exceeds 80–90%, reflecting growing recognition of mental health as a workplace concern [11].

Despite their widespread availability, utilization rates remain persistently low. Reviews across sectors consistently report annual uptake rates between 3% to 8% of eligible employees, with some organizations reporting figures below 1% (4,7). Importantly, low utilization persists even when services are free, externally provided, and formally promoted as confidential. This disconnect between provision and use has been described as a central paradox in workplace mental health interventions [12].

While underutilization statistics document the scale of the problem, they offer limited insight into the mechanisms underlying non-use. Usage rates alone do not explain how employees interpret available services, how work environments shape decision-making, or why formal access does not translate into behavioral engagement. Addressing underutilization, therefore, requires moving beyond descriptive metrics to explore the behavioral processes that sustain non-engagement in everyday work contexts.

### 2.2. Individual-level determinants of utilization

At the individual level, research has identified several factors associated with engagement or avoidance of MSS. Concerns about confidentiality and fear of negative career consequences are among the most consistently reported barriers [4,6]. Even when services are delivered externally, employees often remain uncertain about data privacy and information flows within the organization, undermining trust and the willingness to seek help.

Stigma plays a central role in shaping help-seeking behavior. Studies distinguish between self-stigma, where individuals internalize beliefs that seeking help signals weakness, and anticipated stigma, where employees expect judgment from colleagues or supervisors [6]. Both forms are particularly salient in performance-driven, male-dominated work environments, where emotional resilience is often implicitly valued [4].

Other individual-level factors include perceived need, emotional awareness, and self-efficacy. Employees may normalize stress as an inevitable aspect of work or believe that available services are intended only for severe psychological problems, reducing perceived relevance [13]. However, much of this literature treats help-seeking as an individual choice, often abstracted from the organizational conditions in which decisions are made. As a result, individual perceptions are frequently examined in isolation from the structural and cultural contexts that shape them.

### 2.3. Organizational-level determinants of utilization

Organizational-level research highlights how structural and cultural factors shape employees’ engagement with MSS. Leadership behavior has emerged as a key determinant. When managers actively endorse mental health initiatives or model help-seeking behavior, employees report greater trust and willingness to engage with services [7,14]. Conversely, leadership silence or purely symbolic support can reinforce perceptions that services are peripheral or risky to use.

Workplace culture also plays a decisive role. Norms emphasizing constant availability, long working hours, and self-reliance discourage help-seeking by framing it as incompatible with professional identity [4,8]. Structural constraints such as time scarcity, inflexible schedules, lack of private spaces, and digital access barriers further limit employees’ practical ability to use services during the workday.

Communication strategies influence engagement as well. Generic or compliance-driven wellbeing messaging often fails to resonate with employees’ lived experiences, whereas transparent, empathetic communications that directly address confidentiality concerns are more likely to build trust [4,12]. However, organizational determinants are often examined separately from individual factors, limiting understanding of how structure and culture interact with personal capability and motivation to shape behavior.

### 2.4. Role, identity, and variability in help-seeking

An emerging body of research suggests that engagement with mental-support services varies across professional roles, seniority levels, and occupational identities. Managers may avoid using services to maintain an image of competence and authority, while frontline employees may face greater time constraints and reduced autonomy to seek support during work hours [15]. Employees in compliance- or performance-oriented roles may be particularly sensitive to confidentiality risks and reputational concerns.

Despite these insights, empirical studies that systematically examine how role identity and organizational position shape the use of mental-support services remain limited [16,17]. Non-users, who constitute the majority of employees in most organizations, are especially underrepresented. As a result, existing research offers limited insight into how everyday work practices, role expectations, and identity-related concerns interact to sustain persistent non-engagement.

### 2.5. Theoretical frameworks for behavioral diagnosis

To address the fragmentation of existing findings, behavioral science frameworks offer integrative lenses for understanding help-seeking behavior. The COM-B model, developed by Michie and colleagues [9], provides a comprehensive framework for understanding the determinants of behavior. According to the model, behavior (B) arises from the interaction of three components: Capability (C), Opportunity (O), and Motivation (M). Capability refers to an individual’s physical and psychological capacity to perform a behavior, including relevant knowledge, skills, and mental processes. Opportunity encompasses external conditions that enable or prompt the behavior, spanning both physical opportunities (e.g., access to resources, availability of services) and social opportunities (e.g., workplace culture, managerial support). Motivation captures the cognitive and emotional processes that energize and direct behavior, ranging from reflective processes (e.g., conscious decision-making, weighing benefits and risks) to automatic processes (e.g., habits, stigma-related responses, emotional reactions).

The COM-B model has been widely applied to health-related behaviors, including help-seeking and service uptake, yet its application to workplace mental-support service underutilization remains limited. Few studies explicitly use COM-B to explore sustained non-use among employees who have never accessed available services. This gap is particularly salient in organizational contexts where services are formally available but remain practically inaccessible.

Taken together, the literature highlights a persistent gap between the availability of mental health support services and employees’ behavioral engagement with them. Existing studies document relevant individual, organizational, and cultural factors but often examine these in isolation. There is a clear need for integrative, behaviorally informed research that examines how these factors interact in real work contexts to sustain non-use. The present study addresses this gap by applying the COM-B model to explore the underutilization of mental health support services among non-users in a high-tech organizational setting.

## 3. Methods

### 3.1. Design of the study

This study employed a participatory, exploratory qualitative design to explore the persistent underutilization of MSS in a high-tech organizational setting. The study was guided by the Capability, Opportunity, Motivation–Behavior (COM-B) model [9], which conceptualizes behavior as emerging from the interaction of capability, opportunity, and motivation. COM-B was used as an organizing framework to structure both data collection and interpretation, ensuring that interviews moved beyond general attitudes toward the service and instead captured the behavioral conditions shaping non-engagement in everyday work contexts.

The research design combined semi-structured interviews with co-design prompts embedded in the interviews. This approach enabled the study to generate both (a) in-depth experiential accounts of barriers and facilitators shaping non-use and (b) participant-informed suggestions for redesigning MSS in ways that better align with employees’ daily work realities. The study did not evaluate a specific intervention; instead, it aimed to produce behavioral diagnosis and design-oriented insights to inform future organizational improvements.

### 3.2. Context

The study was conducted in a high-tech organization in Türkiye that had implemented a comprehensive Employee Assistance Program (EAP) offering confidential mental health support. Despite formal availability and organizational communication efforts, annual service uptake remained persistently below 2%. This context provided a critical case for investigating underutilization, as low engagement persisted even under conditions typically assumed to facilitate service use (e.g., no direct cost to employees, formal confidentiality, and established organizational provision). The organization’s work environment was characterized by knowledge-intensive roles and high-performance demands, making it a relevant setting to explore how workload, time pressure, and professional norms shape help-seeking behavior.

### 3.3. Participants

Participants were recruited from a large, high-tech organization that offered free, confidential mental health support services to all employees, despite persistently low uptake. Recruitment was conducted through a general email distributed by the Human Resources department across multiple departments. All employees who received the invitation were eligible to participate; participation was voluntary and uncompensated. Employees who chose to respond contacted the researcher directly, ensuring that the organization did not know who participated.

The sample consisted of 13 employees, all of whom had never previously accessed the organization’s MSS. This reflects the broader organizational context, in which overall service utilization remained extremely low. Focusing exclusively on non-users allowed the study to explore barriers, perceptions, and decision-making processes underlying sustained non-engagement with workplace mental-support services.

Participants represented a range of organizational roles, including individual contributors, team leads, and managerial staff. They were drawn from multiple functional areas, including engineering, finance, operations, sales, marketing, compliance, information technology, customer support, and human resources. Length of employment within the organization ranged from less than one year to over fourteen years, providing insight into how both early-career and long-tenured employees perceived and navigated available support services.

The sample consisted of 13 employees representing a range of demographic and professional backgrounds. Participants ranged in age from 25 to 52 years (M_age_ = 34 years) and included both women and men employees. They worked in diverse functional areas, including engineering, finance, operations, sales, marketing, compliance, information technology, customer support, and human resources. Participants occupied different organizational roles, including individual contributors, team leads, and managerial staff. Organizational tenure ranged from less than one year to more than fourteen years (M = 6.8 years). To protect participant anonymity within the organizational context, more detailed demographic information such as exact ages or department-specific identifiers is not reported. This approach ensured confidentiality while allowing meaningful interpretation of how organizational role, responsibility level, and work context shaped perceptions of, and barriers to, engagement with workplace mental-support services.

### 3.4. Data collection

Data collection was conducted in June 2023 using semi-structured, in-depth interviews designed to explore employees’ perceptions of, and barriers to, engaging with workplace MSS. An interview guide was developed to ensure consistency across participants while retaining flexibility to probe individual experiences in detail. The guide was theory-informed by the Capability, Opportunity, Motivation–Behavior (COM-B) model, which provided a structured lens for examining cognitive, environmental, and motivational factors influencing help-seeking behavior.

Interview questions addressed several domains, including participants’ understanding of the mental-support service, perceived relevance to their work and personal context, organizational and social constraints, emotional and motivational considerations, and reflections on professional identity and vulnerability. Open-ended prompts encouraged participants to elaborate on their experiences and reasoning processes. Example questions included: *“What comes to mind when you think about the mental-support service offered by the organization?”* and *“What makes it difficult or easy to consider using this service within your daily work routine?”* The complete interview guide is provided as Supporting Information (S1 File).

Each interview concluded with a co-design activity to move beyond problem identification toward solution generation. During this phase, participants were invited to propose concrete changes to the design, communication, or delivery of the service; reflect on the feasibility of these ideas within their organizational context; and imagine alternative formats that could better support engagement [18]. Co-design prompts focused on reducing practical barriers, strengthening motivation, and aligning the service more closely with everyday work practices. The complete set of co-design prompts and activities is provided as Supporting Information (S2 File).

Interviews were conducted by the author, a researcher with training in qualitative methods and behavioral science. Depending on participants’ preferences, interviews took place either in person in a private on-site meeting room or via secure video conferencing. Interviews lasted between 33 and 49 minutes. Before each interview, participants were reminded of the voluntary nature of participation, assured of confidentiality, and informed of their right to withdraw at any time without consequence. Written informed consent was obtained from all participants. All interviews were audio-recorded with permission, transcribed verbatim, anonymized, and securely stored in accordance with institutional data protection guidelines.

### 3.5. Data analysis

Data analysis was conducted in a structured, two-stage qualitative process. The first stage focused on exploring barriers and facilitators shaping employees’ engagement with workplace MSS, while the second stage explored participant-generated design suggestions emerging from the co-design component. Throughout both stages, the COM-B model provided an organizing framework for interpretation.

#### 3.5.1. Stage 1: Exploratory analysis of barriers and facilitators.

Interview transcripts were analyzed using reflexive thematic analysis as described by Braun and Clarke [19,20]. This approach was selected because it offers both flexibility and interpretive depth for exploring complex social phenomena. The analysis was abductive, combining deductive elements from the COM-B model with inductive openness to emergent, context-specific themes. Analysis began with repeated readings of the transcripts to establish familiarity with the data. Initial codes were generated to capture participants’ descriptions of their understanding of the service, perceived constraints, emotional responses, and reasoning related to help-seeking at work.

Coding was conducted at both semantic and latent levels, capturing not only explicit statements but also underlying assumptions, tensions, and patterns across participants. Codes were iteratively reviewed and clustered into subthemes, which were subsequently organized within the three COM-B domains. This process enabled systematic identification of psychological and physical capability constraints, physical and social opportunity barriers, and reflective and automatic motivational dynamics influencing non-engagement.

In keeping with the epistemological stance of reflexive thematic analysis, inter-coder reliability was not calculated, as coding was understood as an interpretive act rather than an objective categorization task. Instead, rigor was established through reflexivity, transparency, and collaborative discussion. Coding memos were created for each transcript to track analytic reasoning. Finally, care was taken to select representative and vivid quotations that captured the essence of each theme, while ensuring anonymity.

#### 3.5.2. Stage 2: Analysis of co-design outputs.

Co-design data were analyzed separately from the exploratory interview data to preserve the conceptual distinction between understanding existing barriers and generating potential solutions. Co-design outputs included participants’ proposed ideas, suggested features, and reflections on how workplace mental-support services could be redesigned to better fit their daily work context.

These materials were collated across interviews and subjected to a thematic grouping process focused on identifying recurring design directions rather than individual-level experiences. Design suggestions were examined in relation to their intended function (e.g., reducing cognitive effort, increasing accessibility, reinforcing positive emotional experiences) and then mapped to COM-B domains to assess how they addressed previously identified barriers.

This analytic step did not aim to evaluate or test interventions but to synthesize participant-informed design implications grounded in lived experience. The resulting set of design suggestions is presented in the Results section and summarized in Table 2.

### 3.6. Ethics

This study was reviewed and approved by the Middle East Technical University (ODTÜ) Human Subjects Ethics Committee (Approval No: 0183-ODTÜİAEK-2023). All participants were provided with detailed information about the study’s purpose and procedures before participating. Written informed consent was obtained from all participants prior to their inclusion in the study, in accordance with the ethical standards of the committee and the principles outlined in the Declaration of Helsinki. Participation was entirely voluntary, and participants were informed of their right to withdraw from the study at any time without providing a reason and without any consequences. All data were collected, stored, and analyzed in accordance with institutional ethical guidelines and relevant data protection regulations.

## 4. Results

A total of 524 codes were identified and organized within the COM-B framework (Capability, Opportunity, Motivation). The analysis captured a broad spectrum of behavioral determinants affecting engagement with workplace mental-support services, ranging from informational and cognitive aspects to emotional, environmental, and social dynamics. Representative quotations are presented to illustrate each subtheme.

### 4.1. Capability

#### 4.1.1. Psychological capability.

Psychological capability referred to employees’ cognitive and emotional capacity to recognize needs, process information, and regulate actions related to seeking mental support. Three subthemes were identified: knowledge and understanding, attention and cognitive load, and self-regulation strategies. Knowledge and understanding captured participants’ awareness of available services and how to access them. Limited informational clarity was repeatedly noted:

*“I’ve seen the posters and the link, but I still don’t understand what kind of help it actually gives.”*

Employees reported that unclear language and a lack of examples prevented them from differentiating between programs for crisis intervention and everyday stress management.

*“It’s hard to know if it’s for someone with serious problems or just for regular stress—so I avoid it.”*

Attention and cognitive load reflected the difficulty of engaging with information amid competing mental demands.

*“There’s just too much happening during the day; even reading an email about it feels like extra work.”*

This overload often led to passive disengagement, in which employees acknowledged the availability of support but felt mentally too saturated to act. Self-regulation strategies represented the ability to monitor emotions and initiate adaptive coping. Those who had previously developed emotional-awareness practices were better able to seek help when needed:

*“When I feel myself getting overwhelmed, I stop and check what support I can use—it’s part of my routine now.”*

#### 4.1.2. Physical capability.

Physical capability captured bodily states that enabled or restricted engagement. Subthemes included fatigue and energy state, physical capacity constraints, and fitness and physical limits. Fatigue and energy state emerged as a dominant barrier. Many participants cited tiredness, physical exhaustion, or sleep deprivation as reasons for not attending support sessions:

*“By the end of the day, I’m drained; even a helpful session feels like another task.”*

Physical capacity constraints reflected how ongoing health conditions or recovery periods interfered with mental-support use.

*“After my surgery, I was focusing on physical therapy—mental support just wasn’t a priority.”*

### 4.2. Opportunity

#### 4.2.1. Physical opportunity.

Physical opportunity included material, temporal, and structural resources that either facilitated or impeded engagement. Subthemes were time constraints and scheduling, access to environments and logistics, financial barriers, and tool or device usability and reliability. Time constraints and scheduling were the most frequently cited limitations. Employees struggled to fit sessions into their schedules:

*“All the workshops are during work hours, and you feel guilty leaving your desk.”*

When time and place were not aligned with workflow, participation declined. Access to environments and logistics addressed the physical context for support. Employees described the absence of private spaces or difficulties with digital access:

*“There’s no quiet room where I can talk freely without colleagues overhearing.”*

Financial barriers were occasionally mentioned, particularly for external therapy referrals:

*“The first sessions are free, but then you have to pay. It’s discouraging.”*

Tool or device usability and reliability included references to digital interface issues, technical errors, or distrust in data accuracy:

*“The app keeps freezing—it’s easier to give up than keep trying.”*

#### 4.2.2. Social opportunity.

Social opportunity encompassed interpersonal and cultural conditions that shaped comfort and acceptability in using mental support. Subthemes included peer and team norms and support, family and partner support, professional guidance and expectations, and social norms and pressures. Peer and team norms strongly influenced behavioral modeling.

*“When others in my team joined the program, it became normal to try it.”*

Professional guidance and expectations captured how managerial and organizational signals determined legitimacy.

*“When HR introduced it as a development tool, people felt it wasn’t just for problems—it was for everyone.”*

Social norms and pressures reflected perceived stigma and judgment.

*“People think if you need help, you’re weak. That’s why most don’t sign up.”*

Family and partner support was occasionally mentioned as an external motivator for participation, especially among those encouraged by relatives to seek help.

### 4.3. Motivation

#### 4.3.1. Reflective motivation.

Reflective motivation involved deliberate reasoning processes shaping decisions to engage. Subthemes included goal setting and prioritization, beliefs and self-efficacy, intentions and commitment, and outcome expectations and rationale. Goal setting and prioritization described how individuals aligned mental support with personal or professional objectives.

*“I joined to manage stress better because it affects how I lead my team.”*

Beliefs and self-efficacy reflected confidence in one’s ability to benefit from services.

*“I knew it could help, but I wasn’t sure I could open up and make it work.”*

Tangible results strengthened intentions and commitment:

*“After two sessions, I noticed I was sleeping better—that made me want to continue.”*

Outcome expectations and rationale described how anticipated benefits or doubts influenced choices.

*“I’m not convinced a few conversations can fix real problems.”*

#### 4.3.2. Automatic motivation.

Automatic motivation encompasses habitual and emotional processes operating without conscious planning. Subthemes included affect and emotional tone, habits and cues, reinforcement and rewards, and fatigue and emotional depletion. Affect and emotional tone highlighted the threshold for seeking help.

*“I was too anxious to even open the booking page.”*

Habits and cues described how established work patterns displaced help-seeking:

*“Whenever I feel stressed, I automatically start working more instead of pausing.”*

Reinforcement and rewards referred to positive emotions following participation:

*“After the first session, I felt lighter—it reminded me that it’s worth the effort.”*

Fatigue and emotional depletion further illustrated how chronic stress reduced the capacity for engagement:

*“Sometimes I just shut down. Even small things like signing up feel impossible.”*

These codes underscored that emotional energy directly affected behavioral activation and continuity.

### 4.4. Cross-domain patterns

Interconnections among domains were frequent. Participants who possessed knowledge and awareness (psychological capability), had access to time and privacy (physical opportunity), and experienced emotional relief (automatic motivation) demonstrated the highest engagement levels.

*“Once I understood how easy and confidential it was, I actually started looking forward to sessions.”*

Conversely, when one domain was restricted. For example, due to a lack of time, energy, or trust, behavioral engagement diminished.

*“I know it’s useful, but between deadlines and exhaustion, I can’t make it happen.”*

The coded patterns revealed that successful engagement required the alignment of informational clarity, environmental feasibility, and emotional readiness. Table 1 summarizes the identified COM-B components and associated subthemes, providing an overview of the key behavioral barriers and facilitators influencing engagement with workplace MSS.

**Table 1:** Summary of findings across COM-B components.

COM-B Component	Subthemes	Description
**Psychological Capability**	Knowledge and understanding	Limited clarity about the purpose, scope, and appropriate use of the mental-support service reduced employees’ ability to assess its relevance to their needs.
	Attention and cognitive load	High workload and constant task-switching limited employees’ mental capacity to process information about the service or to initiate help-seeking actions.
	Self-regulation strategies	Variability in employees’ ability to recognize emotional strain and initiate supportive actions influenced readiness to seek help.
**Physical Capability**	Fatigue and energy state	Physical exhaustion and depleted energy reduced perceived capacity to engage with support services, even when motivation was present.
	Physical capacity constraints	Health conditions or recovery demands shifted attention away from mental support toward physical functioning and work continuity.
**Physical Opportunity**	Time constraints and scheduling	Rigid schedules and workload pressures made participation in sessions feel incompatible with daily work routines.
	Access and logistics	Lack of private spaces and friction in digital access hindered the use of confidential and convenient services.
	Financial barriers	Uncertainty about cost coverage beyond initial sessions discouraged continued engagement.
	Tool usability	Technical difficulties and complex interfaces reduced the likelihood of continued access to the service.
**Social Opportunity**	Peer and team norms	Observed behaviors and attitudes within teams shaped perceptions of whether using support services was acceptable or risky.
	Professional guidance and expectations	Signals from managers and HR influenced whether the service was framed as a developmental resource or a sign of weakness.
	Social pressures and stigma	Anticipated judgment and implicit norms of self-reliance discouraged help-seeking behavior.
**Reflective Motivation**	Goal alignment and prioritization	Employees weighed mental-support use against professional goals and often deprioritized it when immediate work demands dominated.
	Beliefs and self-efficacy	Doubts about personal benefit and ability to engage effectively reduced intentional commitment.
	Outcome expectations	Skepticism about the effectiveness of short-term support limited perceived value.
**Automatic Motivation**	Emotional responses	Anxiety, shame, or discomfort acted as affective barriers to initiating engagement.
	Habitual coping patterns	Default responses, such as working longer hours, replaced help-seeking behaviors.
	Reinforcement and emotional depletion	Positive emotional relief supported continued engagement, whereas chronic depletion undermined activation.

### 4.5. Co-Design insights and Participants’ suggestions

In addition to describing barriers and facilitators, participants offered practical ideas for improving the accessibility, usability, and sustainability of workplace mental health support services. These suggestions reflected lived experience and emphasized how design modifications could address gaps identified across Capability, Opportunity, and Motivation.

Participants recommended clearer, more interactive communication to strengthen awareness and understanding.

*“If there was a short explainer video or examples of what sessions look like, more people would try it.”*

Several suggested integrating micro-learning modules or brief digital prompts that build emotion-regulation skills over time.

*“Even small reminders or exercises in Teams could keep the idea of support visible without overwhelming us.”*

Participants emphasized the need for restorative spaces and physical recovery breaks to counteract fatigue.

*“It would help to have a quiet room or recharge space where you can breathe before a session.”*

Others proposed optional movement-based activities (e.g., walking meetings or short stretching breaks) embedded in the workday to increase overall readiness for mental-health engagement.

To improve structural accessibility, employees suggested flexible scheduling, on-demand booking, and hybrid formats.

*“If I could join a 20-minute session between meetings or from home, I’d actually attend.”*

They also requested simplified digital interfaces with single-sign-on access through existing workplace tools:

*“It should be as easy as joining a meeting, no extra logins or forms.”*

Financial transparency was another recurring point; participants wanted clear information about costs, coverage, and available free sessions.

Participants consistently highlighted the role of organizational culture. They proposed visible leadership endorsement, peer-led testimonials, and optional group formats to normalize participation.

*“If managers shared their own experiences, it would feel normal, not like something you hide.”*

Anonymity and confidentiality features were also requested, such as privacy indicators during virtual sessions and non-traceable booking systems.

Participants suggested incorporating goal-tracking and self-reflection tools to connect mental support use with personal progress.

*“It would be motivating to see how sessions affect my stress levels or focus over time.”*

They also valued follow-up messages summarizing achieved outcomes, which reinforced perceived usefulness and commitment.

To encourage positive emotional reinforcement, employees proposed feedback loops that emphasize wellbeing improvements, such as progress badges or instant reflections after sessions.

*“Getting a small message saying ‘you took time for yourself today’ would actually make me smile.”*

They also suggested integrating supportive cues, such as notifications, reminders, or environmental prompts, that nudge engagement without pressure.

*“A gentle nudge once a week works better than constant reminders.”*

Participants’ design suggestions, mapped to COM-B components, are synthesized in Table 2.

**Table 2:** Summary of co-design themes.

COM-B Component	Participants’ Design Suggestions
**Psychological Capability**	Provide clear, concrete explanations of what the service offers; use short visual explainers and examples to reduce ambiguity and cognitive effort.
**Physical Capability**	Introduce restorative spaces and recovery breaks to support energy regulation and readiness for engagement.
**Physical Opportunity**	Offer flexible scheduling, shorter session formats, hybrid or remote access options, and simplified booking through existing workplace platforms.
**Social Opportunity**	Encourage visible leadership participation, share peer experiences, and strengthen privacy safeguards to normalize help-seeking.
**Reflective Motivation**	Integrate goal-setting and progress reflection tools that connect service use to personal and professional outcomes.
**Automatic Motivation**	Embed positive reinforcement mechanisms such as affirming feedback, gentle reminders, and emotionally supportive cues following engagement.

## 5. Discussion

This study explored the persistent underutilization of mental-support services (MSS) within a high-tech organization, despite organizational investment and formal availability. Guided by the Capability, Opportunity, and Motivation–Behavior (COM-B) model, the findings revealed that underutilization is shaped by interacting constraints across psychological capability, physical opportunity, and motivational readiness. Notably, the study focused exclusively on employees who had never accessed the service, addressing a persistent gap in workplace mental health research that has tended to prioritize the experiences of service users or those already engaged in support [7,12]. Beyond behavioral diagnosis, the integration of co-design prompts enabled participants to translate lived barriers into feasible redesign directions, consistent with participatory approaches to service improvement and co-creation, psychological capability, physical opportunity, and motivational readiness.

### 5.1. Capability

The findings expand understanding of capability by showing that informational awareness alone does not reliably translate into behavioral engagement. Participants often recognized that MSS existed but lacked clarity about the service offered, when it was appropriate to use it, and how it would fit their needs. This aligns with prior research showing that awareness and availability are insufficient conditions for uptake when employees remain uncertain about relevance or anticipate negative consequences [3,4,6]. In other words, awareness functioned as a passive state, whereas psychological capability reflected a more active and situated capacity to interpret, decide, and initiate help-seeking under real-world constraints [9].

A particularly salient capability-related barrier was cognitive overload. When employees described high workload intensity and constant task-switching, even low-effort actions, such as opening a booking page or reading service information, were experienced as cognitively costly. This finding aligns with broader evidence that job demands and mental strain can undermine self-regulatory resources and reduce engagement with wellbeing initiatives [1,21]. From a COM-B model perspective, this indicates that psychological capability includes not only knowledge but also the mental capacity to act on that knowledge at the right moment [9]. Practically, MSS communication may therefore need to reduce cognitive effort by using concrete examples, low-friction entry points, and brief, emotionally accessible formats, rather than relying on information-heavy HR channels.

Physical capability barriers further reinforced that embedded states partly shape readiness to engage. Fatigue and depleted energy were described as invisible inhibitors, with employees reporting that even a potentially helpful session could feel like another task after a demanding day. This complements evidence linking high-intensity work contexts to exhaustion and burnout risk, which can reduce employees’ capacity to initiate supportive behaviors [1]. Rather than treating fatigue as an outcome, the present findings suggest it may function as a behavioral bottleneck that constrains engagement even when motivation is present. This implies that increasing uptake may require not only improving access to support but also addressing upstream conditions that restore energy and reduce depletion, such as workload rhythms, recovery opportunities, and time autonomy [2,21].

### 5.2. Opportunity

Opportunity-related barriers highlighted that environmental design often shapes behavior more strongly than intention. Participants repeatedly described time constraints, lack of privacy, and logistical issues as reasons for non-engagement. These findings align with organizational mental health literature, which emphasizes that structural conditions such as time scarcity, inflexible scheduling, and insufficient private space can make formally available services behaviorally inaccessible [3,8,21]. From a COM-B model perspective, this reinforces that opportunity is not simply the presence of a service, but the degree to which physical and social environments enable action in practice [9].

Participants’ co-design suggestions directly targeted these opportunity constraints through practical redesign proposals, including shorter formats, flexible scheduling, hybrid access, and simplified booking integrated into existing workplace tools. These ideas align with established service design principles that aim to reduce friction and increase accessibility by integrating workflows and providing low-threshold entry points [22]. They also reflect the broader recommendation that workplace mental health initiatives must be embedded into organizational systems rather than treated as optional add-ons competing with core work demands [2,21].

Social opportunity findings underscored the behavioral power of workplace norms and leadership signals. Stigma was not always explicit, but it was embedded in subtle expectations of uninterrupted productivity and self-reliance. This is consistent with evidence that anticipated stigma and fear of reputational consequences reduce help-seeking in workplace contexts [6,13]. Participants described leadership silence as an implicit signal that mental support is peripheral or risky, whereas visible managerial endorsement was perceived as a legitimizing cue. This aligns with research showing that leaders can shape psychological safety and the perceived acceptability of using mental health resources [7,14]. Importantly, the present findings suggest that leadership modelling may function as a scalable permission structure, reducing perceived risk without requiring immediate structural overhaul, although structural constraints still remain critical.

Collectively, opportunity-related findings indicate that underutilization should not be attributed solely to individual reluctance. Instead, it reflects environments designed for uninterrupted output rather than human variability in stress, attention, and emotional readiness. Improving uptake, therefore, requires redesigning the system conditions that make help-seeking feasible, safe, and compatible with everyday work practices [2,21].

### 5.3. Motivation

The findings indicate that motivation to engage with MSS is not a linear outcome of awareness or intention, but an interplay between reflective evaluation and automatic emotional dynamics. This distinction is consistent with the COM-B model’s separation of reflective and automatic motivation [9] and is supported by workplace stigma research, which shows that help-seeking decisions often involve both careful risk-benefit considerations and affective avoidance responses [6,13].

Reflective motivation was shaped by beliefs about usefulness, confidentiality, and identity congruence. Participants were more likely to consider engagement when MSS could be interpreted as supporting performance sustainability rather than signalling vulnerability. This resonates with organizational literature, which emphasizes that help-seeking is strongly shaped by professional identity and perceived legitimacy within the work culture [4,8]. Conversely, skepticism about organizational sincerity, doubts about confidentiality, or uncertainty about outcomes reduced perceived control and undermined intention. These patterns align with prior findings that trust and perceived psychological safety influence whether employees view workplace support as a credible and personally safe option [6].

Automatic motivation operated through emotional feedback loops and habitual coping patterns. Participants described avoidance driven by anxiety, shame, or discomfort, as well as default responses such as working more when stressed. This aligns with evidence that stigma-related affect and avoidance processes can suppress help-seeking even when individuals recognize potential benefits [6]. At the same time, positive affective experiences following engagement were described as reinforcing and sustaining. This supports broader theoretical accounts suggesting that positive emotional experiences can broaden coping repertoires and build psychological resources over time [23]. Practically, this implies that MSS should not only be accessible and rationally convincing but also emotionally safe and rewarding to use. Small reinforcement mechanisms, such as affirming feedback, brief reflections, or supportive follow-up cues, may help shift engagement from a one-time action to a repetitive practice.

### 5.4. Co-Design

Integrating co-design allowed the study to move from behavioral diagnosis toward actionable redesign insights grounded in employee experience. Participants’ suggestions mapped directly onto the three COM-B model domains: clarifying service scope and use cases (capability), reducing friction through flexible formats and workflow integration (opportunity), and supporting identity-safe engagement and reinforcement (motivation). This convergence is consistent with participatory design research showing that users can generate context-sensitive solutions when invited to articulate needs and feasibility constraints [18,22].

Rather than positioning co-design as a tested intervention, the present study uses co-design as a structured mechanism for eliciting redesign directions. This is methodologically valuable because it supports translation from qualitative insights to practical design requirements while preserving the lived context that produced non-use. In organizational wellbeing settings, such participatory approaches are increasingly recommended to ensure interventions are feasible, acceptable, and aligned with everyday work practices [2,21]. In this study, co-design outputs also served as a credibility check: employees’ proposed solutions directly targeted the behavioral mechanisms identified in the interview analysis, suggesting coherence between the diagnostic and redesign directions.

### 5.5. Design implications

The findings suggest several actionable directions for organizations aiming to increase engagement with MSS. First, MSS communication should be designed for cognitive simplicity by using concrete examples, short visual explainers, and minimal-effort entry points that reduce decision ambiguity and cognitive load. Second, services should be structurally integrated into everyday workflows through flexible scheduling, shorter formats, and low-friction access embedded in platforms employees already use. Third, organizations should strengthen social permissions by encouraging visible leadership endorsement and normalizing help-seeking as compatible with professional competence. Fourth, design should account for automatic motivation by making engagement emotionally safe and reinforcing, for example, through supportive follow-up cues, affirming feedback, and privacy-protecting interaction patterns. Collectively, these strategies move beyond traditional awareness campaigns toward behavioral ecosystem design, where supportive actions become feasible within work rhythms, socially legitimate, and emotionally rewarding.

### 5.6. Limitations and future directions

This study has several limitations that should be considered when interpreting the findings. First, the study is based on qualitative data from a relatively small sample of thirteen employees within a single high-tech organizational setting in Türkiye. As a result, the findings should not be interpreted as broadly generalizable across organizations, sectors, or national contexts. Instead, the results provide an in-depth, contextually grounded understanding of how behavioral factors may sustain the non-use of workplace mental-support services in a specific organizational environment. Second, the sample included only employees who had never accessed MSS, which strengthens insights into persistent non-use but limits direct comparison with service users. Third, while co-design outputs provide practical redesign directions, the study did not implement or evaluate these suggestions. Future research should test whether redesign strategies that reduce friction and increase social permission translate into measurable changes in uptake, and whether motivational reinforcement mechanisms predict sustained engagement over time. Longitudinal mixed-method studies could further examine how changes in workload rhythms, leadership signaling, and perceived confidentiality shape help-seeking trajectories.

## 6. Conclusion

This study explored the persistent underutilization of workplace mental-support services (MSS) through the lens of the Capability, Opportunity, and Motivation–Behavior (COM-B) model in a high-tech organizational setting. The findings indicate that non-use is sustained through interacting constraints across capability, opportunity, and motivation. Employees’ non-engagement was shaped by uncertainty about service relevance and timing, and also by workload-driven time scarcity, limited privacy, and identity-sensitive concerns that made help-seeking feel risky or impractical in everyday work life.

By integrating semi-structured interviews with co-design prompts embedded in the interviews, the study provides a behaviorally grounded account of underutilization and translates employee experiences into actionable redesign directions. These insights suggest that improving MSS uptake requires moving beyond awareness-based approaches toward organizational designs that reduce friction, strengthen social permission, and better align support with employees’ daily work realities. Future research should evaluate whether such redesign strategies lead to measurable improvements in engagement and sustained use over time.

## Supporting information

S1 File Semi-structured interview guide used in the study. The file contains the complete set of interview questions developed to explore employees’ perceptions, barriers, and decision-making processes related to workplace mental-support services, structured according to the COM-B framework.
(DOCX)

S2 File Co-design prompts used during interviews. The file includes the set of prompts and activities used in the co-design phase of the interviews to elicit participants’ suggestions for improving the design and delivery of workplace mental-support services.
(DOCX)
