# Supplementary material for: From exploration to co-design: Understanding and redesigning workplace mental-support services
Source: PLoS One. 2026 May 8;21(5):e0348067. doi: 10.1371/journal.pone.0348067 (PMC13155620; doi:10.1371/journal.pone.0348067)
Supplement: S1 File — The file contains the complete set of interview questions developed to explore employees’ perceptions, barriers, and decision-making processes related to workplace mental-support services, structured according to the COM-B framework. (DOCX) [file pone.0348067.s001.docx]

**Semi-Structured Interview Guide**

**Purpose:** This interview guide was designed to explore employees’ perceptions, experiences, and behavioral drivers regarding engagement with workplace mental health support services. The guide was informed by the Capability, Opportunity, Motivation–Behavior (COM-B) model. It was used flexibly to allow participants to elaborate on issues most salient to their own work context.

**Section 1. Introduction and Context**

- Can you briefly describe your role and how long you have been working in this organization?
- How would you describe a typical workday for you?

**Section 2**

- What comes to mind when you think about the mental health support service offered by the organization?
- How did you first hear about this service?
- How clear is it to you what the service is intended for and how it works?
- Are there aspects of the service that feel confusing or unclear?

**Section 3**

- How do workload, deadlines, or daily pressures affect your ability to think about or engage with mental-support resources?
- Are there moments when seeking support feels mentally complicated or overwhelming?
- Can you describe situations where you felt you might benefit from support but did not pursue it?

**Section 4**

- How easy or difficult would it be for you to use the service during your workday?
- What practical factors (e.g., time, scheduling, location, privacy) influence your decision?
- How does the organizational environment or culture affect whether people feel comfortable using such services?
- How do managers or colleagues talk about mental health or support, if at all?

**Section 5**

- How do you feel about the idea of using mental support services in relation to your professional role?
- What concerns, if any, might discourage you from using the service?
- What would make the service feel more relevant or worthwhile to you?
- Do you think using such services aligns or conflicts with expectations in your role or team?

**Section 6**

- Is there anything else you think is essential to understand about why people do or do not use these services?
- Is there anything about your experience that we have not discussed but you feel is relevant?
