# Supplementary material for: From exploration to co-design: Understanding and redesigning workplace mental-support services
Source: PLoS One. 2026 May 8;21(5):e0348067. doi: 10.1371/journal.pone.0348067 (PMC13155620; doi:10.1371/journal.pone.0348067)
Supplement: S2 File — The file includes the set of prompts and activities used in the co-design phase of the interviews to elicit participants’ suggestions for improving the design and delivery of workplace mental-support services. (DOCX) [file pone.0348067.s002.docx]

**Co-Design Prompts and Activities**

**Purpose:**

The co-design component was used to move beyond identifying barriers toward generating participant-driven solutions. Participants were invited to propose concrete ideas for improving accessibility, usability, and engagement with workplace mental health support services, drawing on their lived experience within the organization.

**Co-Design 1: Reducing Barriers**

- If you could change one thing about the current mental-support service, what would it be?
- What makes the service feel difficult or inconvenient to use?
- What could be removed or simplified to make engagement easier?

**Co-Design 2: Improving Fit with Daily Work Practices**

- How could the service better fit into your daily workflow?
- What formats (e.g., shorter sessions, flexible timing, digital options) would make participation more realistic?
- Where and when would it feel most natural to access support during a workday?

**Co-Design 3: Motivation and Emotional Experience**

- What would make using the service feel more positive or rewarding?
- What kinds of feedback, follow-up, or reinforcement would encourage continued use?
- How could the service help people feel supported rather than judged?

**Co-Design 4: Organizational Culture and Communication**

- How should the organization discuss mental health support services so people take them seriously?
- What role should managers or leaders play in encouraging engagement?
- Would peer stories or shared experiences help normalize use? Why or why not?

**Co-Design 5: Ideal Future Scenario**

- Imagine the mental health support service worked perfectly for you. What would it look like?
- How would you know that it was helping?
- What would be different in the organization if this service were widely used?
